# UV-Enhanced Artificial Synapses Based on WSe_2_-SrAl_2_O_4_ Composites

**DOI:** 10.3390/nano15241890

**Published:** 2025-12-17

**Authors:** Qi Sun, Xin Long, Chuanwen Chen, Ni Zhang, Ping Chen

**Affiliations:** Center on Nano-Energy Research, Guangxi Key Laboratory for Relativistic Astrophysics, School of Physical Science and Technology, Guangxi University, Nanning 530004, China; qisun@st.gxu.edu.cn (Q.S.); longxin@st.gxu.edu.cn (X.L.); chuanwenchen@st.gxu.edu.cn (C.C.); zhangni@st.gxu.edu.cn (N.Z.)

**Keywords:** artificial synapse, ultraviolet light, WSe_2_-SrAl_2_O_4_, radiation reabsorption

## Abstract

Optoelectronic synapses based on transition metal dichalcogenides have received much attention as artificial synapses due to their good stability in the air and excellent photoelectric properties; however, they suffer from ultraviolet light-triggered synapses due to the ultraviolet insensitivity of transition metal dichalcogenides. In this paper, an ultraviolet-enhanced artificial synapse was achieved on WSe_2_ combined with SrAl_2_O_4_: 6% Eu^2+^, 4% Dy^3+^ phosphor. The strong ultraviolet absorption of SrAl_2_O_4_: 6% Eu^2+^, 4% Dy^3+^ phosphor and radiation reabsorption are responsible for the ultraviolet-enhanced response of the WSe_2_-SrAl_2_O_4_ synapse. The excitatory post-synaptic current of the WSe_2_-SrAl_2_O_4_ synapse triggered by a single pulse at 365 nm was enhanced 4 times more than that from 2D WSe_2_, while the decay time of the post-synaptic current was 9.7 times longer than those from the WSe_2_ device. The excellent ultraviolet sensitivity and decay time promoted the good regulation of the synaptic plasticity of the WSe_2_-SrAl_2_O_4_ device in terms of power densities, pulse widths, pulse intervals, and pulse numbers. Furthermore, outstanding learning behavior was simulated successfully with a forgetting time of 25 s. Handwritten digit recognition was realized with 96.39% accuracy, based on the synaptic weight of the WSe_2_-SrAl_2_O_4_ synapse. This work provides a new pathway for ultraviolet photoelectric synapse and brain-inspired computing.

## 1. Introduction

The development of artificial intelligence and big data has placed new demands on computing power. Traditional computers using the Newman system cannot meet current needs, due to the separation of computation and storage [1]. Brain-like artificial neural networks have been proposed as a breakthrough approach, due to their low energy consumption, parallel processing capabilities for data computation and storage, and their memory properties with 10^11^ neurons and 10^15^ synapses [2,3]. Thus, more attention has been paid to the simulation of artificial synapses and neurons. Among all of the materials, two-dimensional (2D) materials have attracted much attention, due to their advantages of atomic layer thickness and low energy consumption [4,5]. In particular, transition metal dichalcogenides (TMDCs) have become core materials for artificial synapse simulation because of their good stability in the air and excellent photoelectric properties [6,7,8,9,10].

Visible light-triggered artificial synapses of TMDCs were first developed and implemented in a MoS_2_/PTCDA hybrid heterojunction [11], where the type-II heterojunction enabled the carriers to be injected, separated, and captured in the interface, along with a high facilitation ratio of 500% for the optoelectronic synapses. Then, visible light-triggered artificial synapses sparked a research boom, based on the visible response of TMDCs themselves or those constructed from heterogeneous structures, such as MoS_2_ [12], WSe_2_ [13], WS_2_ [14], MoS_2_/MoTe_2_ [15], Ta_2_NiS_5_/MoS_2_/Gr [16], and WSe_2_/Gr [17]. In these studies, van der Waals heterostructures have emerged as a pivotal platform for high-performance optoelectronic devices [18,19,20,21,22], owing to their tunable interfacial barriers, highly controllable band alignment engineering, and exceptional optoelectronic coupling capabilities [23,24]. This flexible stacking configuration enables the integration of diverse functional materials, overcoming the limitations inherent in single-component systems. It facilitates not only enhanced photodetection but also complex neuromorphic functionalities, such as high-sensitivity, wavelength-discriminative, and multimodal sensing [25,26,27], thereby laying the foundation for advanced artificial visual systems. Compared to the lush and vibrant visible synapses, little attention has been paid to ultraviolet (UV)-triggered synapses, due to the insensitive response of TMDCs to UV light (Table S1) [28,29].

In 2018, Guo et al. first reported photonic potentiation and electric habituation in a monolayer MoS_2_ with excitation at 310 nm [30]. Four years later, Roy et al. reported the MoS_2_ artificial optoelectronic synapses triggered at 300 nm, where gate-tunable potentiation was achieved with the number of UV pulses as high as 512 [31]. Subsequently, Jo et al. confirmed the weak response of UV pulses in trilayer MoS_2_, MoSe_2_, MoTe_2_, and WSe_2_, where the linearly enhanced synapse potentiation was excited at 400 nm [32]. Then, UV-sensitized materials were utilized to construct the heterojunctions between TMDCs to attain UV artificial synapses [33,34]. ZnO/WSe_2_ and ZnO/MoS_2_ were explored to establish UV artificial synapses for the large absorption cross section and ultrasensitive UV response of ZnO [33,34]. The paired pulsed facilitation (PPF) of ZnO/MoS_2_ optoelectronic synapses was improved to 160% triggered by 375 nm [33]. Later, the heterojunction of GaN/WSe_2_ was built to extend the response range of optoelectronic synapses to the UV range, giving a high image recognition accuracy of 96.6 [35]. Although significant progress was achieved in the UV photo-synapse of TMDCs, they are still in their infancy.

Herein, UV-enhanced response artificial optoelectronic synapses have been created on WSe_2_-SrAl_2_O_4_ composites, where SrAl_2_O_4_: 6% Eu, 4% Dy is responsible for responding to UV light for its large UV absorption section. SrAl_2_O_4_: 6% Eu, 4% Dy transfers UV to visible light centered at 522 nm by the downshift process, followed by absorption and photoelectric conversion in WSe_2_. The UV response was improved to 4 times higher than that from 2D WSe_2_. The photo-synapse performance was obtained, including excitatory post-synaptic current (EPSC), short-term memory (STM), long-term memory (LTM), and the transition from short-term memory (STM) to long-term memory (LTM), excited by 365 nm with various power densities, pulse widths, pulse intervals, and pulse numbers. The simulation of the learning–forgetting–relearning process was imitated with a forgetting time as long as 22 s. Furthermore, the optoelectronic synapses based on WSe_2_-SrAl_2_O_4_ composites can simulate recognition of handwritten digit images with a high accuracy of 96.39%. The study provides a simple approach for UV-enhanced optoelectronic synapses, suggesting the high potential in neuromorphic computing.

## 2. Materials and Methods

### 2.1. Materials Synthesis

In this experiment, layered WSe_2_ was prepared via physical vapor deposition (PVD) in a tube furnace under atmospheric pressure (Figure S1). WSe_2_ powder (99.9%, Alfa Aesar, Shanghai, China) was used as the precursor, and a silicon wafer (Lijing Electronics Co., Ltd., Shenzhen, China) with a surface oxide layer served as the substrate. The detailed procedure was as follows. First, the silicon wafer was cleaned. Then, a quartz boat loaded with WSe_2_ powder was placed at the center of the heating zone, while another quartz boat carrying the silicon substrate was positioned in the deposition zone near the tube mouth in the low-temperature region, with a distance of 8–15 cm maintained between them. Before the reaction, the chamber was evacuated to remove impurities, followed by the introduction of high-purity argon (99.999%, Wangzhou Gas Co., Ltd., Nanning, China) to establish an inert growth atmosphere. During the growth stage, the precursor was heated to the set temperature via a temperature-control system, causing a solid-to-vapor phase transition. The resulting vapor was transported by argon carrier gas to the substrate surface in the low-temperature zone, where layered WSe_2_ was deposited. After growth, the sample was cooled inside the furnace, and its morphology was preliminarily characterized using optical microscopy.

By systematically optimizing the key parameters such as the growth temperature, holding time, and gas flow rate, the optimal process conditions were determined as follows: growth temperature 1185 °C, holding time 5 min, and gas flow rate 30 sccm. Under these conditions, layered WSe_2_ samples with uniform morphology suitable for subsequent device fabrication were successfully obtained.

### 2.2. Device Fabrication

The device fabrication primarily involved two key steps: electrode preparation and SrAl_2_O_4_ powder (SrAl_2_O_4_: 6% Eu^2+^, 4% Dy^3+^, Longli Technology Co., Ltd., Shenzhen, China) coating. The specific procedure was as follows:

1. Electrode preparation and curing. First, silver paste (01H-1803, Sryeo Electronic Paste Co., Ltd., Shenzhen, China) electrodes are precisely applied on both sides of the WSe_2_ material using a probe. The device is then cured at 70 °C for 2 min to ensure stable and reliable contact between the electrodes and the material.
2. SrAl_2_O_4_ coating and post-treatment. SrAl_2_O_4_ powder is dissolved in ultrapure water and ultrasonically dispersed for 5 min to form a homogeneous suspension. The suspension is spin-coated onto the device surface at 1000 rpm for 60 s, using a spin coater. Finally, the device is baked at 100 °C for 1 min to remove the residual moisture. After confirming the uniformity of the coating under an optical microscope, the final WSe_2_-SrAl_2_O_4_ composite device is obtained.

### 2.3. Characterization

The synthesized 2D WSe_2_ was characterized through a suite of microscopic and spectroscopic techniques. The morphology was examined by optical microscopy (OM, Olympus BX43F, Shinjuku City, Japan) and atomic force microscopy (AFM, Bruker Dimension Icon, Billerica, MA, USA). Raman and photoluminescence (PL) spectra were performed on a Horiba iHR550 confocal Raman spectrometer (Horiba, iHR550, Loos, France) with 532 nm laser excitation.

### 2.4. Measurements

The electrical characterization was performed using a semiconductor parameter analyzer (Keithley 4200A-SCS, Solon, OH, USA) equipped with a probe station (Lake Shore CRX-VF, Westerville, OH, USA).

## 3. Results and Discussion

**Figure 1:**
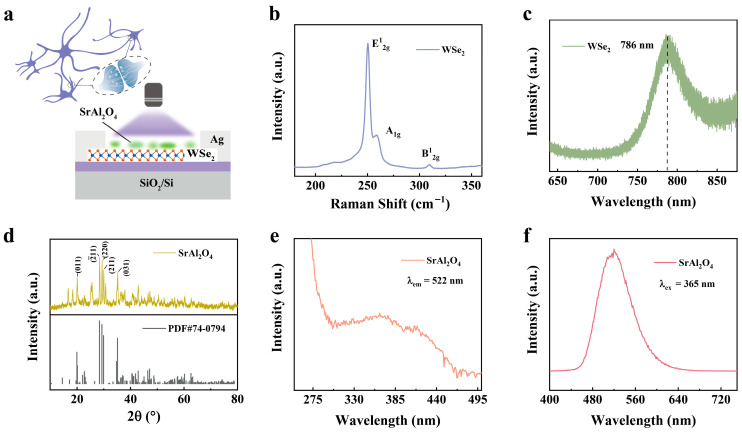
Structure and optical properties of WSe_2_-SrAl_2_O_4_ device. (**a**) Schematic structure of the WSe_2_-SrAl_2_O_4_ device. (**b**) Raman spectrum of WSe_2_. (**c**) PL spectrum of WSe_2_. (**d**) XRD patterns of the SrAl_2_O_4_: 6% Eu^2+^, 4% Dy^3+^ phosphor. Excitation (**e**) and emission (**f**) spectra of the SrAl_2_O_4_: 6% Eu^2+^, 4% Dy^3+^ phosphor.

A UV-enhanced artificial optoelectronic synapse was constructed with WSe_2_ as the electronic synapses and SrAl_2_O_4_: 6% Eu, 4% Dy as the UV-responsive layer (Figure 1a and Figure S2). Layered WSe_2_ was grown by physical vapor deposition (PVD) [36]. The Raman spectrum of WSe_2_ presented three peaks centered around 250 cm^−1^, 260 cm^−1^ and 309 cm^−1^, which are ascribed to E^1^_2g_ and A_1g_ modes and the interlayer interactions B^1^_2g_ of 2H WSe_2_, respectively (Figure 1b). The atomic force microscopy (AFM) image shows the thickness of 2H WSe_2_ is 1.6 nm (Figure S3), indicating bilayer WSe_2_ for this optoelectronic synapse, consistent with the interlayer interactions B^1^_2g_. The luminescence spectra showed a peak at 786 nm (Figure 1c), agreeing with the bandgap of bilayer 2H WSe_2_ [37]. The XRD patterns of the SrAl_2_O_4_: 6% Eu^2+^, 4% Dy^3+^ phosphor were matched very well with the patterns of the monoclinic phase (Figure 1d), revealing the pure phase of SrAl_2_O_4_: 6% Eu^2+^, 4% Dy^3+^ phosphor. The specific parameters are listed in Table S2. Excitation spectra of the SrAl_2_O_4_: 6% Eu^2+^, 4% Dy^3+^ phosphor were detected, which provided a broadband luminescence centered around 380 nm (Figure 1e), suggesting the absorption potential of broadband UV for electronic synapses [38]. The luminescence spectra of SrAl_2_O_4_: 6% Eu^2+^, 4% Dy^3+^ phosphor displayed a broad band around 522 nm [39], fitting well with the absorption of 2H WSe_2_ and implied an enhanced UV response in WSe_2_-SrAl_2_O_4_ optoelectronic synapses (Figure 1f).

**Figure 2:**
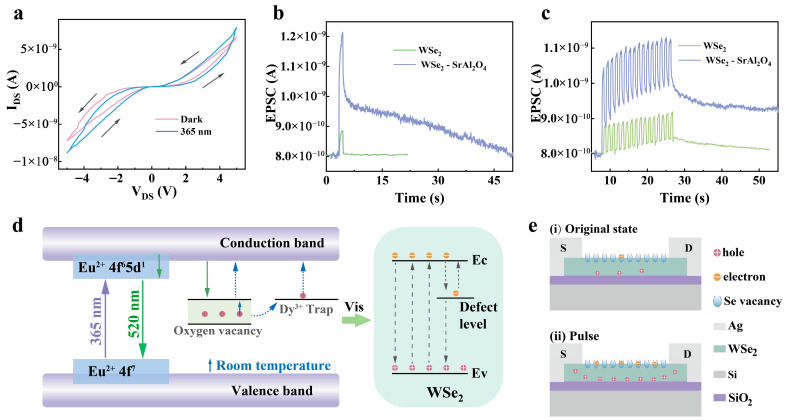
Output curves and EPSC of WSe_2_-SrAl_2_O_4_ device excited by 365 nm. (**a**) Output hysteresis curves of the WSe_2_-SrAl_2_O_4_ device with and without excitation at 365 nm. (The arrows indicate the voltage sweep direction.) (**b**) EPSC generated by single pulse in WSe_2_ and WSe_2_-SrAl_2_O_4_ devices excited at 365 nm with the power intensity (P) at 7.2 mW/cm^2^ and pulse width (W) at 1 s. (**c**) EPSC evoked by a sequence of pulses in WSe_2_ and WSe_2_-SrAl_2_O_4_ devices with pulse numbers (N) at 16 (λ = 365 nm, P = 7.2 mW/cm^2^, W = 0.5 s, Δt = 0.5 s). (**d**) Microscopic mechanism of UV-enhanced photoresponse of WSe_2_-SrAl_2_O_4_ device. (**e**) Working mechanism of WSe_2_-SrAl_2_O_4_ device and the electron capture by Se vacancies or defects at the interface between WSe_2_ and SiO_2_ under (**i**) initial state and (**ii**) single pulse.

The output curve of the WSe_2_-SrAl_2_O_4_ device was first measured to evaluate the UV synaptic potential (Figure 2a). The output curve showed a nonlinear characteristic, indicating Schottky contact between the WSe_2_ and Ag electrodes. The corresponding energy-level diagram of the WSe_2_/Ag contact is provided in Figure S4. The loop scan of the source–drain voltages showed a hysteresis window, revealing the potential of synaptic memory due to incompletely released trapped electrons in traps of 2H WSe_2_ or defects at interface between WSe_2_ and SiO_2_ [40]. Upon excitation at 365 nm, the output curve of the WSe_2_-SrAl_2_O_4_ device exhibited a larger enhanced current and a large hysteresis window, originating from the strong absorption of SrAl_2_O_4_: 6% Eu^2+^, 4% Dy^3+^ phosphor and radiation reabsorption from SrAl_2_O_4_: 6% Eu^2+^, 4% Dy^3+^ to WSe_2_ (Figure 2d). The UV photons were absorbed by SrAl_2_O_4_: 6% Eu^2+^, 4% Dy^3+^ and pumped the electrons from the valence band to the conduction band upon excitation at 365 nm, followed by relaxing to the 5d state of Eu^2+^ by multiphonon relaxation. The populated 5d state of Eu^2+^ gave a green luminescence band around 522 nm with the transition from the 5d to 4f state of Eu^2+^. The green luminescence was reabsorbed by WSe_2_ and pumped the electrons of WSe_2_ from the valence band to the conduction band, resulting in a larger photogenerated carrier concentration and enhanced current. The large hysteresis window was also originated from the larger current, which suggest that defect states captured a large number of electrons and that there was a long release time of the captured electrons. Therefore, the larger enhanced current and hysteresis window of the output curves of WSe_2_-SrAl_2_O_4_ device suggested a good UV synaptic potential.

To confirm the UV synaptic performance, the EPSC of the WSe_2_ and WSe_2_-SrAl_2_O_4_ devices were assessed via triggering by single and multi-pulses (Figure 2b,c). When excited by a single pulse at 365 nm with the power intensity (P) at 7.2 mW/cm^2^ and pulse width (W) at 1 s, an EPSC was generated, because the photogenerated carriers, accompanied by parts of electrons, were captured in the defects in WSe_2_ or the interface between WSe_2_ and SiO_2_ (Figure 2e). After removing the pulse, the EPSC reduced rapidly first and then relaxed to its initial state after 4.7 s. The rapid reduction is connected to the fast recombination of photogenerated carriers. The slow relaxation is ascribed to the tardy release of captured electrons. What is exciting is that the WSe_2_-SrAl_2_O_4_ device presented a higher EPSC and a slower decay time when excited by the same pulse (Figure 2b). The EPSC was almost 4 times higher than that from 2D WSe_2_ owing to the super-sensitive absorption of the SrAl_2_O_4_: 6% Eu^2+^, 4% Dy^3+^ phosphor and radiation reabsorption from SrAl_2_O_4_: 6% Eu^2+^, 4% Dy^3+^ to WSe_2_. The decay time was explored to 45.6 s, which is almost 9.7 times longer than those from a WSe_2_ device, and originated from the long persistence of SrAl_2_O_4_: 6% Eu^2+^, 4% Dy^3+^. At the same time, the enhanced EPSC and decay time were obtained by a sequence of pulses in the WSe_2_-SrAl_2_O_4_ device compared to the WSe_2_ device (Figure 2c), which reconfirmed the ability to regulate the synaptic plasticity.

**Figure 3:**
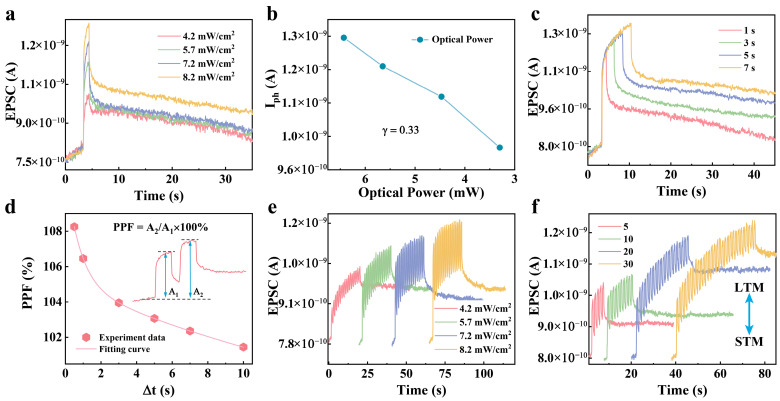
UV synaptic plasticity of the WSe_2_-SrAl_2_O_4_ device. (**a**) EPSC triggered by a single 365 nm pulse (W = 1 s) at varying power densities. (**b**) Variation in photocurrent with optical power. (**c**) EPSC modulated by a single 365 nm pulse with different widths at a power density of 7.2 mW/cm^2^. (**d**) PPF index as a function of pulse interval. Illustration: EPSC response to paired pulses (P = 7.2 mW/cm^2^, W = 1 s, Δt = 0.5 s). (**e**) LTP induced by 16 consecutive optical pulses at different power densities (W = 0.5 s, Δt = 0.5 s). (**f**) EPSC triggered by 5, 10, 20, and 30 light pulses (P = 5.7 mW/cm^2^, W = 0.5 s, Δt = 0.5 s).

The synaptic plasticity of the WSe_2_-SrAl_2_O_4_ device was modulated by power densities, pulse widths, pulse intervals, and pulse numbers (Figure 3). The EPSC increased significantly with increasing power densities from 4.2 mW/cm^2^ to 8.2 mW/cm^2^ and pulse widths from 1 s to 7 s (Figure 3a,c). The growing EPSC was ascribed to the augmented photogenerated carriers, which also induced a larger number of captured electrons in defects. In addition, the slower release of captured electrons in defects after removing the pulse led to a longer recovered time with increasing power densities and pulse widths. Further, the photocurrent obtained at different optical powers in Figure 3a was fitted using a power-law model, yielding a weak capture coefficient (γ) [41] and establishing a relationship between responsivity (R) and optical power [42]. The calculated γ ≈ 0.33 (<1) confirms the presence of significant trap effects in the device (Figure 3b), while R also shows an increasing trend with optical power (Figure S5). This occurs, because at low optical power, carriers are captured by defects, resulting in lower responsivity. As the optical power increases, the traps gradually become saturated, which improves the carrier transport efficiency and, correspondingly, enhances the responsivity. This mechanism is consistent with that described in Figure 2. Then, the PPF index was measured with varying pulse intervals (Figure 3d). The PPF index was calculated as [43]

(1) $$\mathrm{PPF}\;\mathrm{index}=\frac{A_{2}}{A_{1}}\;\times \;100\%,$$

where A_1_ and A_2_ represent the EPSCs triggered by the first and second pulses, respectively. The PPF index can be fitted by a double-exponential function [44]:

(2) $$\mathrm{PPF}\;\mathrm{index}\;=\;C\;+{\;B}_{1}\exp\left(-\frac{\Delta t}{\tau_{1}}\right)\;+\;B_{2}\exp\left(-\frac{\Delta t}{\tau_{2}}\right)$$

where C is a constant, B_1_ and B_2_ correspond to the initial magnitudes of rapid and slow decay, and τ_1_ and τ_2_ are the respective relaxation time constants. Fitting of the results shows 0.80 s and 20.65 s for time constants τ_1_ and τ_2_, respectively. The order-of-magnitude difference between the two time constants is similar to the synaptic performance of organisms [45]. In addition, the synaptic plasticity of the WSe_2_-SrAl_2_O_4_ device was also regulated successfully by multiple pulses with different pulse counts and densities (Figure 3e). Further, the transition from STM to LTM was realized easily by increasing the pulse count from 5 to 30 at 365 nm (Figure 3f). The gradual decay of EPSC after the termination of the UV pulses represents an optical habituation or erasing process of the synaptic weight, analogous to the natural forgetting behavior observed in biological synapses.

**Figure 4:**
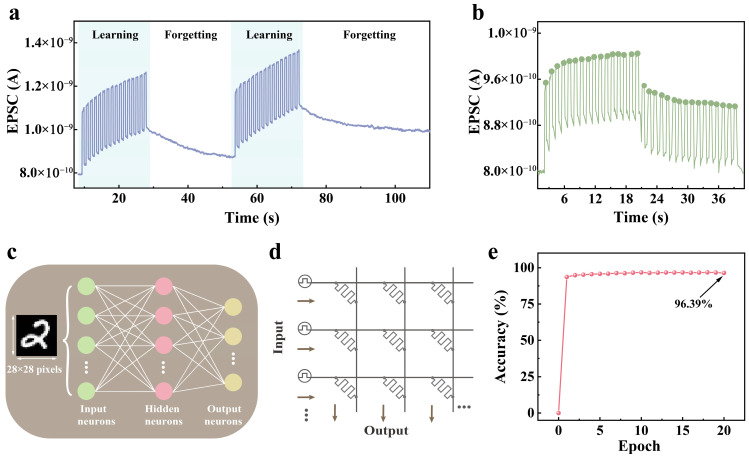
Human learning behavior and handwritten digit recognition by ANN-based behavior WSe_2_-SrAl_2_O_4_ synaptic weights. (**a**) Learning behavior simulated by 365 nm light pulses (P = 5.7 mW/cm^2^, W = 0.5 s, Δt = 0.5 s). (**b**) Conductance changes modulated by 532 nm and 365 nm (P = 5.7 mW/cm^2^, W = 0.5 s, Δt = 0.5 s). (**c**) Schematic of the three-layer ANN used to recognize MNIST images. (**d**) Cross-switch array structure. (**e**) Recognition accuracy from the MNIST simulation for each training.

Furthermore, human learning behavior was also simulated based on the WSe_2_-SrAl_2_O_4_ UV synapse (Figure 4a). The first learning was imitated by 16 pulses, with the EPSC increasing to 1.3 × 10^−9^ A from 7.9 × 10^−10^ A. Then, the forgetting process began and lasted for 25 s, followed by a relearning process with the same triggering conditions. When the seventh pulse was applied, the EPSC rose to 1.3 × 10^−9^ A, which was the same outcome as for the first learning process. After continuous triggering with nine subsequent pulses, the EPSC rose to 1.4 × 10^−9^ A, similar to relearning reinforcement behavior. Later, the forgetting process began again. After a 35 s re-forgetting process, the EPSC still remained higher than the first forgetting value, analogous to the forgetting time of an organism. The learning–forgetting–relearning sequence clearly illustrates the habituation behavior of the WSe_2_-SrAl_2_O_4_ synapse. The EPSC increases during the first learning and then decreases during the subsequent forgetting period. Subsequently, the EPSC recovers more rapidly during relearning, indicating that the device can both store and gradually erase synaptic information. Furthermore, the synaptic behavior of the WSe_2_-SrAl_2_O_4_ device can be modulated by 532 nm and 365 nm light illumination. As shown in Figure 4b, 16 pulses of 532 nm light (P = 5.7 mW/cm^2^, W = 0.5 s, Δt = 0.5 s) were applied first, followed by 16 pulses of 365 nm light (P = 5.7 mW/cm^2^, W = 0.5 s, Δt = 0.5 s). These results, combined with the modulation of the synaptic behavior by visible light shown in Figure S6, collectively demonstrate that the device can achieve active non-destructive optical synaptic weight erasure in addition to the passive forgetting mechanism.

A three-layer artificial neural network (ANN) was built (Figure 4c) to evaluate the neuromorphic computing capability of the device, consisting of 784 input neurons, 300 hidden neurons, and 10 output neurons. The synaptic weights between layers were extracted from LTP/LTD characteristics and implemented in a crossbar-array simulator (Figure 4d labeled point). When tested on the MNIST dataset, the network achieved a recognition accuracy of 96.39% after 20 training epochs (Figure 4e), demonstrating the device’s potential for practical neuromorphic classification tasks.

## 4. Conclusions

A UV-enhanced artificial optoelectronic synapse was achieved in a WSe_2_-SrAl_2_O_4_ device. SrAl_2_O_4_: 6% Eu^2+^, 4% Dy^3+^ phosphor was used to sensitize UV light and transfer to visible light by a downshift process, followed reabsorbing visible light by 2H WSe_2_. The EPSC of the WSe_2_-SrAl_2_O_4_ synapse was increased 4 times more than that from 2D WSe_2_, owing to the super-sensitive absorption of SrAl_2_O_4_: 6% Eu^2+^, 4% Dy^3+^ phosphor. The decay time was explored to 45.6 s, which is almost 9.7 times longer than with the WSe_2_ device. The excellent UV sensitivity and decay time promoted the good regulation of the synaptic plasticity of the WSe_2_-SrAl_2_O_4_ device by power densities, pulse widths, pulse intervals, and pulse numbers. In addition, the transition from STM to LTM was achieved easily by increasing the pulse width to 7 s or the pulse number to 20. Perfect human learning behavior was realized, with a forgetting time as long as 25 s. Furthermore, the synaptic weight of the WSe_2_-SrAl_2_O_4_ device was applied to recognize handwritten digits with the accuracy as high as 96.39%. Our strategy provides a new road for a UV optoelectronic synapse based on TMDCs and presents a potential application in neuromorphic computing.

## Data Availability

The original contributions presented in this study are included in the article and Supplementary Materials. Further inquiries can be directed to the corresponding author.

## Supplementary Materials

The following supporting information can be downloaded at https://www.mdpi.com/article/10.3390/nano15241890/s1, Figure S1: Schematic of WSe_2_ growth system by PVD method; Figure S2: Optical image of WSe_2_-SrAl_2_O_4_ device; Figure S3: Characterization of 2D WSe_2_ materials AFM (a) and SEM (b); Figure S4: Band structure diagram of WSe_2_/Ag; Figure S5: Variation in responsivity with optical power; Figure S6: Synaptic plasticity of the WSe_2_ devices triggered by 532 nm; Table S1. Comparison of TMDCs-based UV optoelectronic synapses with this work; Table S2. Microstructural parameters of sample SrAl_2_O_4_: 6% Eu^2+^, 4% Dy^3+^ [46].
