# Supplementary material for: UV-Enhanced Artificial Synapses Based on WSe2-SrAl2O4 Composites
Source: Nanomaterials (Basel). 2025 Dec 17;15(24):1890. doi: 10.3390/nano15241890 (PMC12735879; doi:10.3390/nano15241890)
Supplement: Supplementary file 1 [file nanomaterials-15-01890-s001.zip › nanomaterials-4020774-supplementary.pdf]

nanomaterials

MDPI

Article

UV-enhanced artificial synapses based on WSe_2-$SrAl_2O_4$ composites

Qi Sun, Xin Long, Chuanwen Chen, Ni Zhang, Ping Chen *

Center on Nano-Energy Research, Guangxi Key Laboratory for Relativistic Astrophysics, School of Physical Science and Technology, Guangxi University, Nanning 530004, China; qisun@st.gxu.edu.cn (Q.S.); longxin@st.gxu.edu.cn (X.L.); chuanwenchen@st.gxu.edu.cn (C.C.); zhangni@st.gxu.edu.cn;

* Correspondence: chenping@gxu.edu.cn

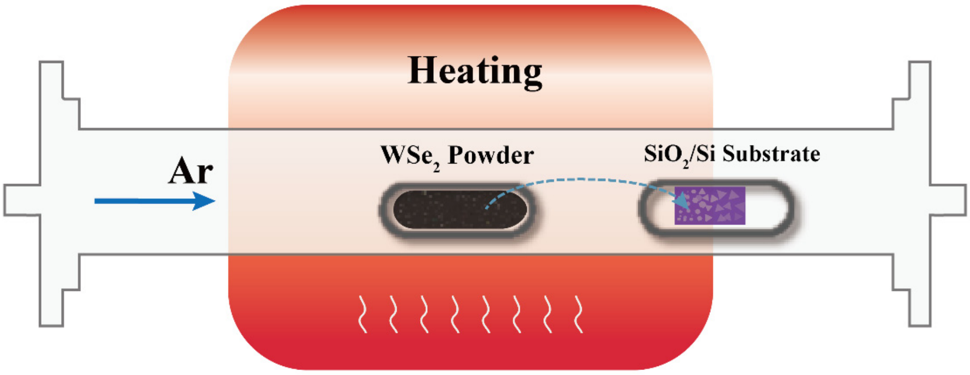

Figure S1. Schematic of WSe2 growth system by PVD method.

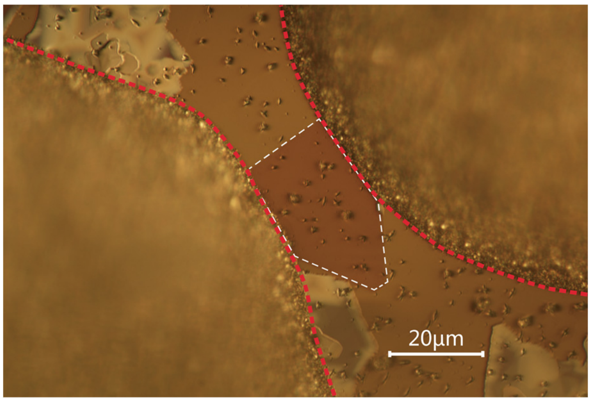

Figure S2. Optical image of WSe_2-$SrAl_2O_4$ device. The effective active area (WSe_2-$SrAl_2O_4$) and Ag electrodes are marked by white and red dashed lines, respectively.

 https://doi.org/10.3390/xxxxx

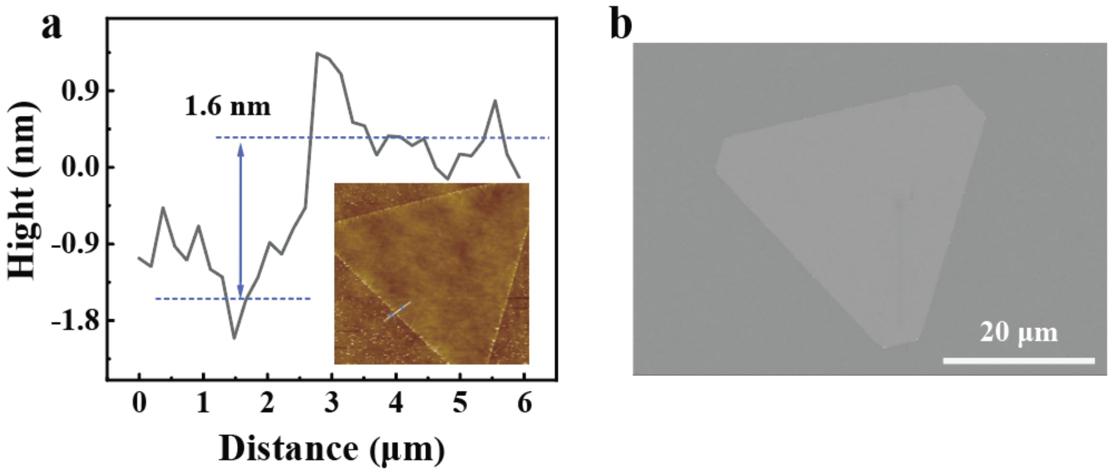

Figure S3. Characterization of 2D WSe_2 Materials AFM(a) and SEM(b).

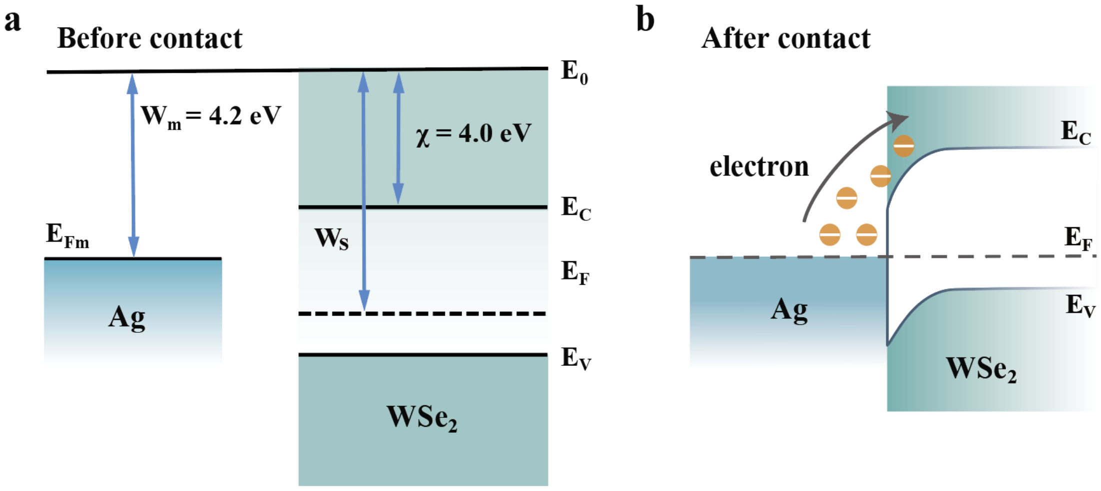

Figure S4. Band structure diagram of WSe_2/Ag. (a) Energy levels before contact, showing the Ag work function (4.2 eV), WSe_2 electron affinity (4.0 eV), and a bandgap of 1.59 eV. (b) After contact, electron injection induces band bending and forms a Schottky barrier.

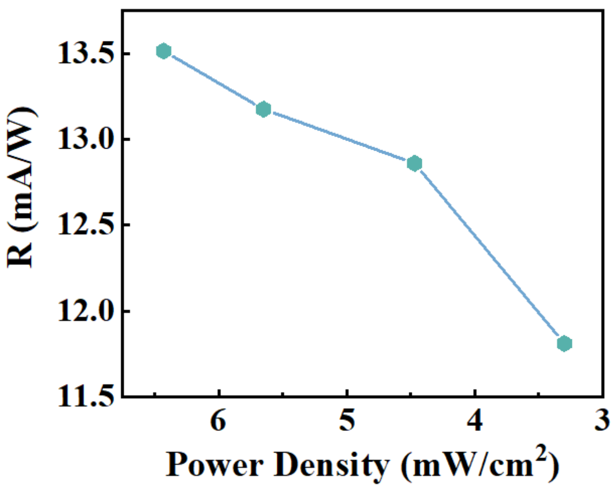

Figure S5. Variation of responsivity with optical power.

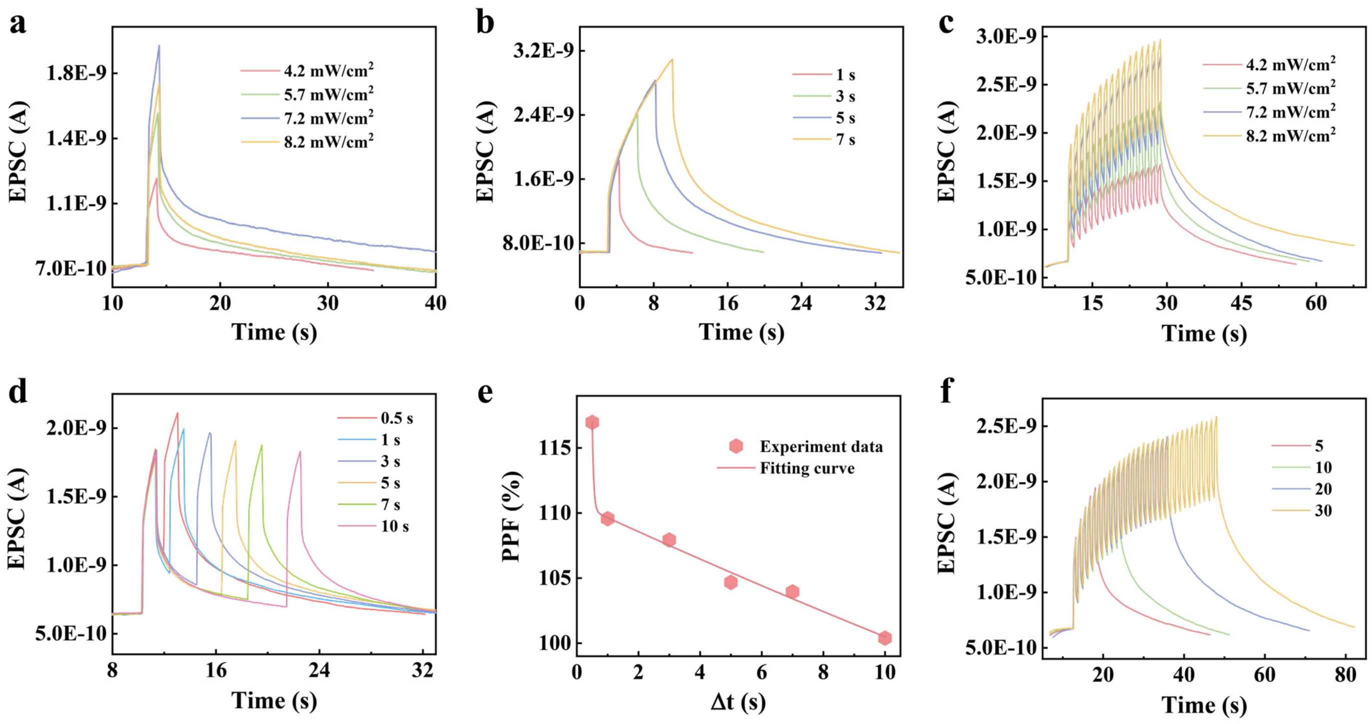

Figure S6. Synaptic plasticity of the WSe_2 devices triggered by 532nm. (a) EPSC triggered by a single 532 nm pulse (W = 1 s) at varying power densities. (b) EPSC modulated by a single 532 nm pulse with different widths at a power density of 7.2 W/cm^2. (c) LTP induced by 16 consecutive optical pulses at different power densities (W = 0.5 s, Δt = 0.5 s). (d) EPSC response induced by paired pulses with different time intervals. (P = 7.2 mW/cm^2, W = 1 s, Δt = 0.5 s). (e) PPF index as a function of pulse interval. (f) EPSC triggered by 5, 10, 20 and 30 light pulses (P = 5.7 mW/cm^2, W = 0.5 s, Δt = 0.5 s).

Table S1. Comparison of TMDCs based UV optoelectronic synapses with this work.

Device Structure	Excitation Wavelength	Reported Power Consumption	Mechanism	Key performance	Limitations/Notes
MoS_2 [1]	310 nm	~1.5mJ	Intrinsic UV absorption	Photonic potentiation + electric habituation	Requires high-energy UV; limited response
$MoS_2/PtTe_2$ [2]	300 nm	~10 nJ	UV-gated modulation	Potentiation with up to 512 pulses	High UV power: no visible-light operation
MoS_2, $MoSe_2$, $MoTe_2$, and WSe_2 [3]	400 nm	0.014nJ	Weak UV absorption	Linear potentiation	Weak synaptic gain; multilayer sensitivity low
ZnO/MoS_2 heterostructure [4]	375 nm	~1.12 μJ	UV sensitization by ZnO	PPF up to 160%	Needs heterojunction; UV. only device
ZnO/WSe_2 heterostructure [5]	375 nm	~4 pJ	ZnO UV-sensitized	Enhanced UV response	Complex interfaces; limited memory functions
GaN/WSe_2/h-BN heterostructure [6]	365 nm	---	GaN UV enhancement	Handwritten digit recognition 96.6%	UV-only; high-power excitation
This work: WSe_2-$SrAl_2O_4$ composite synapse	365 nm	~10 nJ	Radiation reabsorption	UV response enhanced ×4; handwritten-digit recognition 96.39%	Simple structure; broader spectral usability

Table S2. Microstructural Parameters of Sample $SrAl_2O_4$: 6%Eu^{2+}, 4%Dy^{3+}.

(*hkl*)	2θ (deg)	FWHM (deg)	*D* (nm)	ε (×10^{-3})	σ (GPa)	δ (×10^{14} m^{-2})
(011)	20	0.16	50.6	3.06	191.5	3.91
(-211)	28.4	0.16	50.0	2.16	135.2	4.00
(220)	29.4	0.16	49.8	2.09	130.6	4.03
(211)	30.0	0.18	44.6	2.34	146.3	5.03
(031)	35.2	0.21	3.7	2.10	131.1	6.8

The average crystallite size D was first estimated using the Scherrer equation [7],

$$D = \frac{0.9\lambda}{\beta cos\theta}$$

Whereλ is the X-ray wavelength, β is the full width athalf maximum (FWHM) of the diffraction peak (in radians) after correction for instrumental broadening, and θ is the Bragg angle. To separate the contributions from size and lattice strain, we further employed the Williamson-Hall (W-H) method by plotting $\beta cos\theta$ versus 4 $sin\theta$.

$$\beta cos\theta = \frac{0.9\lambda}{D} + 4\varepsilon\, sin\theta$$

Where the intercept provides the size term and the slope yields the microstrain ε. The stress σ was then evaluated from $\sigma = \frac{E\cdot\varepsilon}{2}$, where E is the Young's modulus, taken as 125 GPa. The dislocation density δ was then evaluated from $\delta = \frac{1}{D^2}$.The calculated values of D, ε, σ and δ for the main diffraction peaks are summarized in the newly added Table S1 in the Supporting information. These results confirm that the $SrAl_2O_4$: 6%Eu^{2+}, 4%Dy^{3+} phosphor exhibits a well-crystallized monoclinic phase with moderate microstrain and dislocation density, which is beneficial for stable long-persistent luminescence and, consequently, for the reliable UV sensitization behavior in the WSe_2-$SrAl_2O_4$ synaptic devices.

References

1. He, H. K.; Yang, R.; Zhou, W.; Huang, H.-M.; Xiong, J.; Gan, L.; Zhai, T.-Y.; Guo, X. Photonic Potentiation and Electric Habituation in Ultrathin Memristive Synapses Based on Monolayer MoS_2. *Small* **2018**, *14*, 1800079, doi:10.1002/smll.201800079.

2.. Islam, M.M.; Krishnaprasad, A.; Dev, D.; Martinez-Martinez, R.; Okonkwo, V.; Wu, B.; Han, S.S.; Bae, T.-S.; Chung, H.-S.; Touma, J.; et al. Multiwavelength Optoelectronic Synapse with 2D Materials for Mixed-Color Pattern Recognition. *ACS Nano* **2022**, *16*, 10188–10198, doi:10.1021/acsnano.2c01035.

3.. Moon, G.; Min, S.Y.; Han, C.; Lee, S.; Ahn, H.; Seo, S.; Ding, F.; Kim, S.; Jo, M. Atomically Thin Synapse Networks on Van Der Waals Photo-Memtransistors. *Adv. Mater.* **2023**, *35*, 2203481, doi:10.1002/adma.202203481.

4.. Li, Z.; Zou, G.; Xiao, Y.; Feng, B.; Huo, J.; Peng, J.; Sun, T.; Liu, L. MoS_2/ZnO-Heterostructured Optoelectronic Synapse for Multiwavelength Optical Information-Based Sensing, Memory, and Processing. *Nano Energy* **2024**, *127*, 109733, doi:10.1016/j.nanoen.2024.109733.

5. Li, X.; Wang, S.; Yang, Y.; Xu, S.; Bao, X.; Zhao, L.; Liu, X.; Pan, Z.; Yang, Y.; Su, S.; et al. Photovoltage Junction Memtransistor for Optoelectronic In-Memory Computing. *J. Mater. Chem. C* **2024**, *12*, 12763–12768, doi:10.1039/D4TC03015J.

6. Gao, Z.; Yu, H.; Su, Z.; Luo, Y.; Memon, M.H.; Chen, W.; Kang, Y.; Sun, H. 2D/3D Integrated Phototransistor for Broadband Artificial Synaptic Applications. In Proceedings of the 2024 IEEE Photonics Conference (IPC); IEEE: Rome, Italy, November 10 2024; pp. 1–2.

7. Kumar Litoriya, P.; Kurmi, S.; Verma, A. Structural, Optical, Morphological and Photoluminescence Properties of $SrAl_2O_4$: Dy by Using Urea Fuel Combustion Method. *Mater. Today Proc.* **2022**, *66*, 2044–2049, doi:10.1016/j.matpr.2022.05.487.
